# Natural Radioactivity and Chemical Evolution on the Early Earth: Prebiotic Chemistry and Oxygenation

**DOI:** 10.3390/molecules27238584

**Published:** 2022-12-05

**Authors:** Boris Ershov

**Affiliations:** Frumkin Institute of Physical Chemistry and Electrochemistry, Russian Academy of Sciences, Leninsky pr. 31-4, 119071 Moscow, Russia; ershov@ipc.rssi.ru

**Keywords:** early earth, ocean, radioactivity, radiolysis, chemical evolution, prebiotic molecules, oxygenation

## Abstract

It is generally recognized that the evolution of the early Earth was affected by an external energy source: radiation from the early Sun. The hypothesis about the important role of natural radioactivity, as a source of internal energy in the evolution of the early Earth, is considered and substantiated in this work. The decay of the long-lived isotopes ^232^Th, ^238^U, ^235^U, and ^40^K in the Global Ocean initiated the oxygenation of the hydro- and atmosphere, and the abiogenesis. The content of isotopes in the ocean and the kinetics of their decay, the values of the absorbed dose and dose rate, and the efficiency of sea water radiolysis, as a function of time, were calculated. The ocean served as both a “***reservoir***” that collected components of the early atmosphere and products of their transformations, and a “***converter***” in which further chemical reactions of these compounds took place. Radical mechanisms were proposed for the formation of simple amino acids, sugars, and nitrogen bases, i.e., the key structures of all living things, and also for the formation of oxygen. The calculation results confirm the possible important role of natural radioactivity in the evolution of terrestrial matter, and the emergence of life.

## 1. Introduction

Chemical elements, including unstable radioactive isotopes, are products of stellar evolution. Therefore, natural radioactivity has been an intrinsic component of the natural environment in all stages of Earth’s evolution. The isotopes from the lifetimes which are comparable with the Earth’s age, and which possess high specific activities are of special interest. This refers to heavy elements, ^232^Th, ^235^U, and ^238^U, and a light element, radioactive potassium ^40^K. These elements have long half-lives and high specific radioactivity. The decay of these isotopes served as a potent internal source of energy and, in addition to the gravitational contraction and impacts of falling meteorites, this promoted melting and differentiation of matter and tectonic activity of the early Earth. The subsequent chemical evolution of the Earth, and emergence and development of life on the Earth, also took place under a considerable influence of natural radioactivity. The presence of radioactive elements has always been an integral condition, and a necessary component of the external and internal environment of the Earth.

The evolution of the early Earth, starting from its origin 4.6 Ga ago, proceeded under the radiation of the early Sun. Extensive physico-chemical transformations in the early atmosphere were initiated by hard UV radiation, powerful electric discharges, meteorite fall, high temperature, and volcanic activity. This, apparently, was among the factors that affected the transformation of inorganic components of the early Earth to more complex organic matter. Currently, the existence and development of the biosphere is determined by the presence of molecular oxygen in the atmosphere. However, in an early stage, the planet’s atmosphere contained mainly CH_4_, H_2_O, H_2_, CO_2_, N_2_, NH_3_, nitrogen, and sulfur oxides, which released during the mantle degassing [1,2,3,4]. Anaerobic atmosphere predominated for at least the first two billion years. The appearance of oxygen during the oxygenic photosynthesis in cyanobacteria is attributed to a later period. Before this, simple anaerobic life forms emerged and developed on the Earth for a long time. These forms appeared very early (3.6–3.8 billion years ago), which is evidenced by the biogenic nature of the earliest layered stromatolites found in north-western Australia [5,6,7,8,9,10]. Their appearance was preceded and accompanied by the transformation of inorganic to organic matter and formation of prebiotic molecules. The change in the composition of the Earth’s atmosphere, towards the predominance of oxygen, took place approximately 2.4 billion years ago [1,2,3,4]. This change was called the Great Oxidation Event (GOE). However, it is important to note that, according to geochemical data, traces of oxygen have always been present in the Earth’s atmosphere [11,12,13,14,15]. The sharp change in the atmosphere composition led to mass extinction of previously existing anaerobic life forms and spread of energetically more favorable oxygen-breathing species. 

The transformation of inorganic to organic matter, including the formation of prebiotic compounds, and subsequent transformation of non-living to living matter, are important parts in the appearance of life on the Earth. The simplest biological life appeared and started to develop using this organic protomatter as the nutritious base [16,17,18,19,20,21]. Charles Darwin believed that the primary life spark could appear in a “warm little pond, with all sorts of ammonia and phosphoric salts, light, heat, electricity, &c., present, that a protein compound was chemically formed, ready to undergo still more complex changes.” [22]. In essence, this idea forms the grounds of the known Oparin-Haldane hypothesis, which implies that the first molecules that constituted the earliest cells were slowly self-organized from the primordial soup [23,24]. This soup is considered to contain prebiotic molecules, i.e., molecules that form living matter and have been derived from the molecules of organic precursors. This idea was confirmed in the 1950s by known experiments of Miller and Urey [25]. They reproduced the atmospheric conditions that presumably existed on primitive Earth by subjecting a mixture of water vapor and volcanic gases (CH_4_, NH_3_, and H_2_) to UV light and electric discharge at high temperature (≤100 °C). The formation of a mixture of amino acids and some other prebiotic molecules was established [26,27]. 

Throughout most of the Earth’s history, the existing life forms differed considerably from the species observed today. According to [16,17,18], the first billion years of biological and ecological evolution showed that bacteria and archaea live in oceans containing large amounts of iron, and traces of oxygen. During the next billion years, where the amount of iron in the oceans decreased and the amount of oxygen increased, the metabolic diversity of prokaryotic microorganisms extended aerobic metabolism; while, eukaryotes, hybrid cells with a new and a different organization, further increased the diversity and ecological complexity of the microbial community. In the first two billion years (Hadean and Archean), biogeochemical fundamentals of carbon, sulfur, nitrogen, and phosphorus cycles, that is, microbial processes, which still underlie all Earth’s ecosystems, were created in the early oceans. New genetic and cellular biological features were engrained in the new eukaryotic cells. This finally resulted in the appearance and development of complex multicellular organisms. Thus, from ecological and evolutionary standpoints, the modern highly organized forms of life are products of this far-away world of simple microorganisms of the early Earth. It is very important to pay attention to the fact that the simple organisms that appeared almost 4 Ga ago and are found in Archean rocks were preceded by a very important stage of Earth development, in particular, the transformation of inorganic matter to organic matter. This organic protomatter initiated the development of the simplest biological forms of life, and oxygenation of the atmosphere accelerated the formation of highly organized forms of life. 

In our opinion, the radiation-induced degradation of oxygen-containing substances, first of all, water of the Global Ocean, was an important source of oxygen in the early stage of existence of the Earth where the atmosphere was mainly anaerobic. The radiation also initiated the transformation of inorganic matter into organic matter, and the synthesis of prebiotic molecules. 

This review further develops and substantiates the hypothesis proposed previously [28,29,30,31,32], stating an important role of natural radioactivity in the chemical evolution of the early Earth. The goal is to rationalize and evaluate the radiation mechanism of the formation of organic matter and prebiotic molecules, as well as oxygenation of the hydro- and atmosphere. According to the hypothesis, an important part of the “radiation” mechanism of chemical evolution is radiation-induced transformation of the early Earth matter in the Global Ocean. The decay of radioactive isotopes in the ocean initiated extensive chemical transformations of dissolved compounds. The Ocean served as a ***reservoir*** for components of the early atmosphere and the products of their reactions, and simultaneously as a ***converter*** for radiation-induced reactions. As a result, organic matter and oxygen were formed.

## 2. Natural Radioactivity: Long-Lived Isotopes ^232^Th, ^235^U, ^238^U, and ^40^K

Similarly to stable isotopes, radioactive isotopes appeared as a result of nuclear synthesis of stars of various mass [33]. The decay of isotopes is accompanied by emission of γ-ray or α- and β-particles. Currently, more than 300 radionuclides that were formed simultaneously with the solar system are present on the Earth. They are also permanently formed upon the natural decay of long-lived radionuclides or in nuclear reactions induced by cosmic rays [34,35,36].

Table 1 presents characteristics of the isotopes that made the crucial contribution to the chemical evolution of the early Earth. The calculation of the total energy released during the decay of heavy isotopes ^232^Th, ^235^U, and ^238^U took into account the α- and β-particles energies of all intermediate isotopes of the radioactive family [32]. Among light elements, only ^40^K makes not just a merely significant, but a crucial contribution to the radiation environment of the Earth. It has a long half-life and a high specific radioactivity. The ^40^K isotope (natural abundance of 0.0117 %) decays along two pathways: about 89% of ^40^K atoms undergo β-decay to ^40^Ca, and the rest of ^40^K decays are via capture of an electron from the own electron shell by the nucleus (K electron capture), thus forming ^40^Ar. In the prebiotic era, the radiation level on the Earth was very high. It was supported by the energy released upon the isotope decay, which was described by an exponential law.

N_t_ = N_0_e^−λt^ (1)

where N_0_ and N_t_ are the initial and final (at time t) numbers of atoms of the isotope, and λ is the decay constant equal to $\frac{0.693}{T_{1/2}}$.

**Figure 1:**
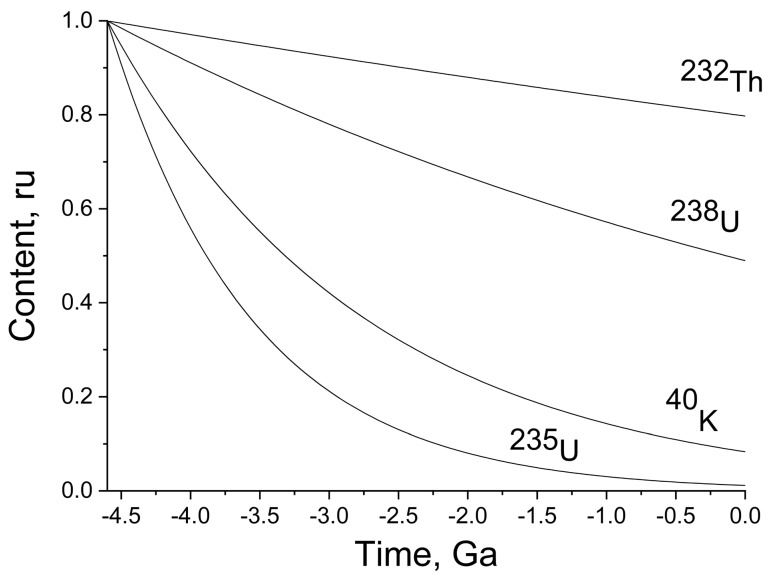
Decay kinetics of the ^232^Th, ^238^U, ^40^K and ^235^U isotopes since the origin of Earth.

The contents of the radioactive elements in the inaccessible Earth’s interior remain unknown. According to [37,38], after differentiation of the Earth’s matter, approximately 77% of radioactive isotopes were concentrated in the Earth’s crust where they occupy the 15–20 km thick near-surface layer. The masses of isotopes in the crust, given in Table 1, were calculated considering the Clarke numbers of elements—the crust mass, which is 2.8 × 10^22^ kg, according to Taylor [38,39], and the known isotope abundances (in %). The corresponding amounts of isotopes in the Global Ocean were calculated [29,32] from their content in sea water [36,40]. The isotope masses 4.6 Ga ago were calculated from Equation (1) and correspond to their fractions retained currently in the Earth’s crust. Figure 1 illustrates the decay kinetics of ^40^K; ^238^U; ^235^U; and ^232^Th from the origin of the Earth to the present time.

The concentration is presented in relative units corresponding to the fraction of the initial amount. The absolute amount (mass) can be easily calculated by multiplying the fraction (Figure 1) by the isotope mass in grams, given in Table 1. The content of ^40^K decreased approximately 10-fold relative to the initial amount. The radioactive isotopes that quantitatively prevail now are those of thorium and uranium (approximately 80% and 50% relative to the initial amounts). 

Table 1 also presents the calculated radiogenic energies released due to decay of radioactive isotopes during 4.6 billion years. The calculation takes into account the decay of isotopes by Equation (1), and includes the energies E_0_ released in one decay event and specific activity λ.

E_r_ = N_0_×E_0_(1 − e^−λt^) (2)

Analysis of the data of Table 1 indicates that the radioactivity of the Earth is concentrated in the crust almost completely. The mass of water in the ocean is 0.02% of the Earth’s mass, and the content of ^40^K, which is the main radiation source, in the ocean is 0.06% of its total amount on the Earth (~77% is in the crust). The greatest contribution to the energy produced by radioactive isotopes on the Earth (1.62 × 10^30^ J) is made by ^40^K. Approximately 36% of the total amount of energy released by now in the crust is caused by the decay of this isotope, while in the ocean this is almost 90%. The relative contents of heavy isotopes in the ocean are largely determined by their solubilities. The thorium solubility is low; therefore, the ^232^Th contribution to the total radioactivity is negligible. The energy released in the crust upon the decay of radioactive isotopes over 4.6 billion years is in total 4.6 × 10^30^ J. Considering the differentiation of the Earth’s matter in the indicated period, this energy should more correctly be referred to the whole Earth’s mass. A greater energy release, amounting to 1.46 × 10^31^ J [41,42], was caused by only gravitational forces. 

**Figure 2:**
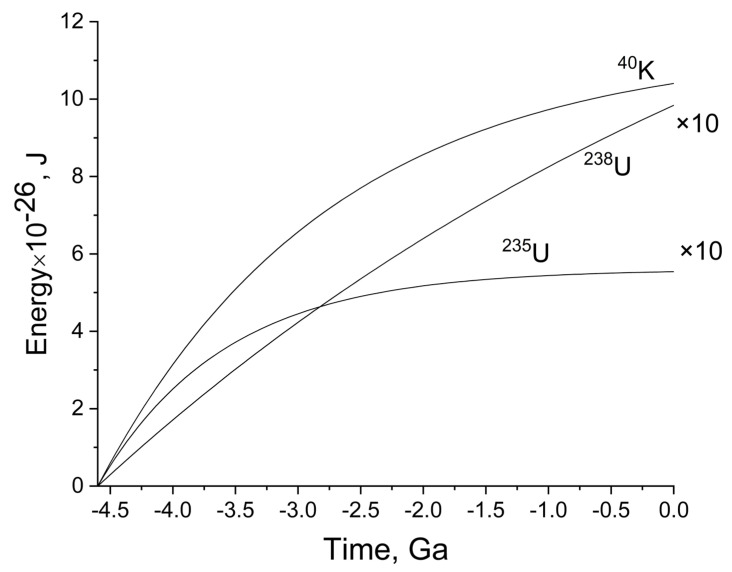
Time dependence of the energy release upon the decay of ^40^K, ^238^U, and ^235^U in the ocean.

Figure 2 shows the time dependence of radiogenic energy release in the ocean. To date, the total value for all isotopes is 1.16 × 10^27^ J. All energy release calculations are based on the assumption that the initial radionuclide is in secular equilibrium with all its decay products, i.e., the activity of all radionuclides that make up the decay series is the same. However, in a complex system of “ocean-host rocks”, a deviation from secular equilibrium is inevitable over billions of years due to geochemical differentiation of isotopes of one series in an environment where some of the intermediate radionuclides are less soluble (mobile) than others. Nevertheless, it can be assumed that a significant part of the radionuclides will not leave the “ocean-host rocks” sphere and will have a radiative effect on water. Therefore, we calculated their possible contribution to the total energy release. It turned out that the contribution of the ^238^U and ^235^U series to the overall energy release pattern is very small, compared to the ^40^K contribution (see Table 1 and Figure 2). Potassium makes a decisive contribution, and the contribution of representatives of the uranium series is approximately 10% or less (if we restrict ourselves to the decay of uranium and some other elements of the uranium series).

The decay of isotopes ^232^Th, ^238^U, ^235^U, and ^40^K also served as a powerful energy source in the early stage of evolution (the first 500 million years). Therefore, it would be reasonable to expect that natural radioactivity has considerably affected the chemical evolution of the Earth: transformation of matter, oxygenation of the atmosphere, and finally the appearance of life.

## 3. Global Ocean and Sea Water Radiolysis

The Global Ocean acted as both a ***reservoir*** for inorganic compounds that entered the ocean from the Earth’s atmosphere and crust, and a ***converter*** for the chemical conversion of compounds induced by the radiation emitted by radioactive isotopes.

### 3.1. Global Ocean

Water has played an important role in the formation of the planet and in the emergence and evolution of life. This is due to quite a number of factors. Among them, noteworthy is the accumulation in the ocean of inorganic compounds and products of their photo- and thermochemical reactions that took place in the atmosphere, and on the surface of the planet. The ocean was a propitious environment for their subsequent physicochemical conversion into organic matter. According to the hypothesis we developed, the crucial role in this process belonged to natural radioactivity [30,32]. This transformation was initiated by the decay of radioactive isotopes. 

Judging by geological and geochemical data, the Earth’s surface oceans have apparently existed from the very early period of Earth’s history, perhaps since the Earth’s origin [43,44,45]. Apparently, the earliest indications of the existence of the hydrosphere on Earth (3.7–4.0 Ga) are volcanoes in the south-western part of Greenland [46], and gneisses in north-western Canada [47] with signs of water-lain sediments. The zircon crystals found in Western Australia [48,49], which might have formed in the presence of water according to isotope and geochemical indications, probably provide evidence for even earlier existence of liquid water on Earth (4.4 Ga). The currently prevailing view is that the Earth has gained most of its water by accretion of carbonaceous chondrite material, particularly CI-like chondrites, from beyond the snow line in the solar nebula [50,51,52,53]. This is supported by the fact that the H/D isotope ratio, in the structurally bound water in carbonaceous chondrites, (140 ± 10) × 10^−6^ is a reasonable match to the terrestrial value. Water in comets is usually characterized by higher D/H ratios, which can be up to 300 × 10^−6^ [54,55], while hydrogen in the solar nebula has D/H ≈ 21 × 10^−6^ [56].

The amount of water in the oceans is 1.4 × 10^21^ kg. The total mass of water in these reservoirs, i.e., besides the ocean water, is estimated at approximately (5–7) × 10^20^ kg [57], i.e., about a half of the ocean’s mass.

### 3.2. Sea water radiolysis

The decay of ^40^K; ^235^U; ^238^U; and ^232^Th initiated the degradation of sea water. The mechanism and energy balance of the interaction of ionizing radiation with water have now been thoroughly studied [58,59,60]. The radiolysis of water can be expressed by the following equation:

H_2_O /\/\/^\^→ e_aq_^−^ (0.28); ^•^H (0.06); ^•^OH (0.28); H_2_O_2_ (0.075); H_2_ (0.045) (3)

The action of γ-ray and high-energy β-particles (fast electrons with energy of ≥1 MeV) gives rise to radical fragments of the water molecule which are highly reactive: hydrated electron e_aq_^−^, hydrogen atom ^•^H, and hydroxide radical ^•^OH; as well as molecular products: hydrogen H_2_, and hydrogen peroxide H_2_O_2_. The values given in parentheses in Equation (3) are the radiation chemical yields of products for the indicated types of radiation (pH 4–9). They are expressed in µmol J^−1^, that is, they are equal to the concentration of particles (in µmol) formed upon the absorption of 1 Joule of radiation energy in 1 kg of water. The yields of e_aq_^−^, ^•^H, and ^•^OH are substantially higher for γ-ray and β-particles than for α-particles, which are mainly generated upon the decay of uranium and thorium. This is due to the fact that for α-particles the concentrations of radicals in the track are very high. Due to recombination, their yields decrease, while the yields of molecular products, on the contrary, increase. Moreover, the action of α-particles, unlike γ-ray and β-particles, gives rise also to the hydroperoxide radical HO_2_^•^. The yields of products of water radiolysis induced by α-particles are: G_(eaq+H)_ = 0.025; G_OH_ = 0.045; G_H2_ = 0.165; G_H2O2_ = 0.155 [58] and G_HO2_ = 0.01 [61] (µmol J^−1^).

Hydrogen peroxide is unstable and tends to decompose

H_2_O_2_ → H_2_O + 1/2 O_2_ (4)

The decomposition of H_2_O_2_ is considerably affected by heat and light, rock catalysis, and the presence of transition metal ions [62]. 

Sea water contains various compounds, first of all, halide ions Cl^−^, Br^−^, and I^−^, which react with the products of water radiolysis. Halide ions catalyze the formation of oxygen in sea water. This is caused by oxidation of chloride ions to hypochlorite ions, which decompose by the reaction [29,30]:

2ClO^−^ → 2Cl^−^ + O_2_ 4 × 10^9^ (5)

Hereinafter, the reaction rate constants are given in L mol^−1^ s^−1^. 

The radiation gives rise, simultaneously, to reactive oxygen species (^•^OH, HO_2_^•^, H_2_O_2_) and species possessing pronounced reducing ability (e_aq_^-^ and H^•^). The standard electrode potentials of e_aq_^−^, ^•^H, and ^•^OH, HO_2_^•^ are −2.9 V, −2.3 V, and +2.8 V, +1.44 V, respectively [63]. The reactions involving these species proceed by a radical mechanism at high rates. The rate constants of e_aq_^-^, ^•^H, and ^•^OH with numerous compounds, including those present in the early Earth’s atmosphere (CH_4_, NH_3_, CO, CO_2_, H_2_S, nitrogen and sulfur oxides, HCN, cyanogen, and many others), were studied by pulse radiolysis and are summarized in a review [59]. These reactions were analyzed in previous studies [29,30,32]. 

Let us note the following important properties of the primary ion-radial products of water radiolysis. The hydrated electron is capable of being transformed to a ^•^H atom via the following reaction:

e_aq_^−^ + H^+^ → ^•^H            2.3 × 10^10^ (6)

As a result, the yield of these species increases, in comparison with the initial yield of ^•^H atoms. In the early period of the Earth’s evolution (Hadeon), the volcanic activity was accompanied by the release of acidic sulfur and nitrogen oxides. Therefore, the features of reactions involving ^•^H were probably significant in the local regions of the planet at that time period. The reactions of ^•^H and ^•^OH with saturated organic compounds (RH) mainly proceed as dehydrogenation

RH + ^•^H (^•^OH) → ^•^R + H_2_ (H_2_O) (7)

while reactions with unsaturated compounds proceed as addition

RH + ^•^H (^•^OH) → ^•^RH_2_ (^•^RHOH) (8)

An important feature of the radiation-induced reactions of compounds dissolved in sea water is that these reactions proceed by a radical mechanism involving radical ion products of water radiolysis, i.e., the action is indirect.

The absorbed dose rates caused by the decay of isotopes are low, with the large total absorbed doses being due to long time of irradiation. The crucial contribution is made by the decay of ^40^K. The absorbed dose rate 4.6 Ga ago for potassium-40 was approximately 1.3 × 10^−10^ Gy s^−1^, while those for ^235^U and ^238^U were 1.2 × 10^−12^ Gy s^−1^ and 0.7 × 10^−12^ Gy s^−1^, respectively [32]. The dose rate decreases by law (1) according to the decay constants λ for these isotopes. As a result, the absorbed dose rate has currently decreased, approximately to 1.0 × 10^−11^ Gy s^−1^ for ^40^K and to 1.0 × 10^−14^ Gy s^−1^ and 0.35 × 10^−12^ Gy s^−1^ for ^235^U and ^238^U, respectively. The absorbed doses increase over time according to Equation (2), being currently 7.1 × 10^5^ Gy for ^40^K, and approximately 1.0 × 10^5^ Gy for the sum of ^235^U and ^238^U [32].

## 4. Radiation-Induced Transformation of the Earth’s Matter: Formation of Organic Molecules

The advent of life on Earth was preceded by transformation of inorganic substances into organic matter, and formation of prebiotic molecules. The chemical evolution of the primary matter of the planet required a source of energy to sustain chemical reactions. It is generally accepted that the chemical transformation of matter, during the first 500 million years after the formation of Earth, was initiated by hard solar radiation, intense electric discharges, fall of meteorites, and volcanic activity. This provided the conversion of simple inorganic compounds containing carbon, nitrogen, sulfur, phosphorus, and other to more complex organic molecules. The arising primary organic molecules were subsequently utilized as primary “structural elements” for the formation of more complex molecules, and/or as the nutrient medium for bacteria (prebiotics). However, the presence of radioactivity on the Earth means the existence of also an internal, powerful, and permanent energy source; the important role of which, in the chemical evolution of the planet, is still to be evaluated. The radical and ionic products of water radiolysis in the ocean could have served as “activators” of inorganic matter conversion into organic. This is evidenced by their high reactivity towards inorganic compounds containing carbon, nitrogen, and sulfur present in the atmosphere and the hydrosphere of the early Earth [30]. The new radicals containing C, N, P, and S atoms, which were formed in the reactions, underwent subsequent radical chain reactions and chain termination reactions upon recombination, and thus formed the organic matter of the planet. Thus, the Global Ocean functioned as a ***converter*** of inorganic compounds to organic matter using the energy released upon the decay of radioactive isotopes. This process, which lasted for hundreds of millions of years, was apparently an important constituent of the Earth’s chemical evolution. 

**Figure 3:**
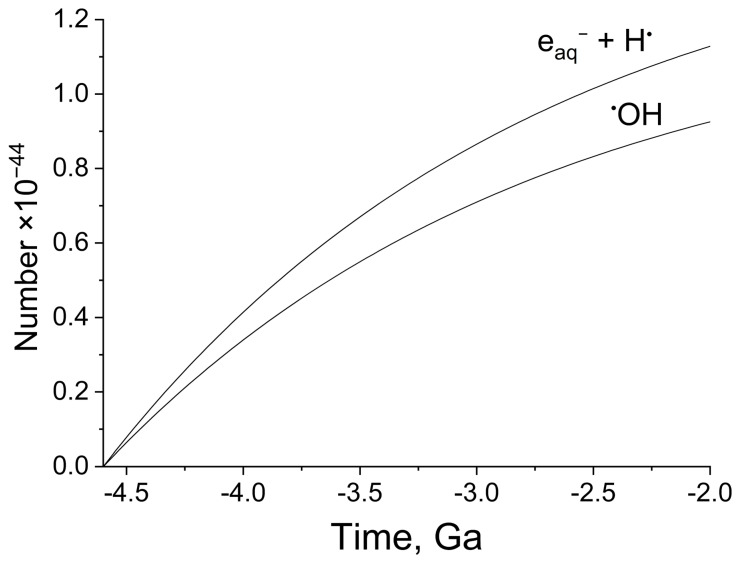
Time dependence of the number of radical species in sea water formed upon decay of isotopes ^40^K, ^235^U, and ^238^U.

The amount of the resulting radicals was sufficiently high to considerably influence the chemical composition of sea water. Indeed, calculations show that by the time of the Great Oxidation Event, i.e., 2.5 Ga ago, the number of radicals formed by the radiation mechanism in the ocean were approximately 2 × 10^44^ species (Figure 3). This was about 0.5 mol of species per liter of water. It is clear that this amount of active species should have markedly affected the chemical evolution of the planet.

## 5. Radical Mechanism of Formation of Amino Acids and Sugars

A generally accepted view is that hydrogen cyanide (HCN) and carbon oxide (CO) served as the sources of carbon and nitrogen to manufacture building blocks from amino acids, nucleotides, sugars, and lipids. There is every reason to define these molecules as “God’s molecules.” These compounds and their derivatives have an unsaturated bond and, as a consequence, they tend to undergo condensation and polymerization reactions, giving rise to prebiotic molecules, and organic matter as a whole. 

HCN was formed in an atmosphere containing N_2_ and rich in methane (CH_4_) or acetylene (C_2_H_2_), but it could also arise in considerable amounts (>1 ppm) in a CO-dominated atmosphere [64]. A necessary condition was the absence of oxygen or, more precisely, low oxygen content in the atmosphere (C/O > 1). The reactions of these gases to give HCN require drastic conditions. HCN appeared by the photochemical mechanism involving the action of hard solar radiation [65,66], high-energy cosmic rays [67], lightning [68,69], and meteorite falls [70]. 

Over the last 20 years, the classical Miller-Urey method, for the synthesis of prebiotic molecules [25,26,27], has been markedly extended by analogous syntheses under various drastic conditions of high-energy impacts in the presence of HCN and formamide (HCONH_2_), resulting from HCN hydration [70,71,72,73,74,75,76,77,78,79,80,81]. HCN is a perfect “chemical” block for the construction of prebiotic complex molecules. Owing to the presence of a triple bond, HCN tends to bind other molecules with unsaturated bonds to form organic matter under the action of excess energy [82]. This situation, mimicking the harsh conditions of the early Earth’s atmosphere, was implemented in the synthesis of prebiotics according to the Miller-Urey mechanism. However, the radiation-induced synthesis proceeds at ambient temperature via the chemical reactions of dissolved compounds present among volcanic gases, and the products of their reactions [28,29,30,31,32]. A specific feature of this process is that the reactions involve free radicals formed upon the radiolysis of water. In other words, the transformation of inorganic molecules is not directly induced by exposure to high-energy radiation (light, plasma, electric discharge, mechanical shock, etc.), but occurs indirectly by a radical mechanism. Currently, many reactions that represent the intermediate steps of this transformation have been studied in sufficient detail by pulsed radiolysis; the reaction rate constants and the nature of the final products have been measured. The results of these studies are summarized in review [59], and in publications [30,32]. Radical reactions are characterized by very high-rate constants. As an example, consider some important reactions involving water-derived radicals that are formed in the ocean upon the decay of radioactive isotopes [59]:

CH_4_ + ^•^OH → ^•^CH_3_ + H_2_O        1.1 × 10^8^ (9)

CO + ^•^OH → ^•^CO_2_H        2 × 10^9^ (10)

NH_3_ + ^•^OH → ^•^NH_2_ + H_2_O        9.7 × 10^7^ (11)

H_2_S + ^•^OH → HS^•^ + H_2_O        1.5 × 10^10^ (12)

HCN + ^•^OH → HOCH=N^•^        6 × 10^7^ (13)

HCHO + ^•^OH → ^•^CHO + H_2_O        ~1 × 10^9^ (14)

H_2_PO_4_^−^ + ^•^OH → OH^−^ + H_2_PO_4_^•^        2 × 10^4^ (15)

CO + •H → •CHO        3.3 × 107(16)

HCN + ^•^H → HN=C^•^H        3.7 × 10^7^ (17)

HCHO + ^•^H → ^•^CHO + H_2_        5.0 × 10^6^ (18)

CO + *e*_aq_^−^ (H^+^) → ^•^CO^−^ (^•^CHO)        1.6 × 10^9^ (19)

CO_2_ + *e*_aq_^−^ → CO_2_^•−^ (^•^CO_2_H)        7.7 × 10^9^ (20)

(CN)_2_ + *e*_aq_^−^ → (CN)_2_^•−^        2.1 × 10^10^ (21)

HCHO + *e*_aq_^−^ → ^•^CH_2_OH + OH^−^        ~1 × 10^7^ (22)

It is evident that these, and many other radiation-induced reactions, took place in the ocean in the early stage of Earth’s existence. These reactions are of interest since they generate fragment radicals which are combined with each other (with termination of the radical state) to give a set of diverse and important organic “molecules of life”, such as amino acids, sugars, and nucleotides. The radical recombination gives a set of organic molecules of the first level of complexity. The subsequent participation of these species in secondary reactions may give larger molecules and/or even macromolecules. Consider, in particular, the mechanism of formation of the simplest amino acid—glycine. This important molecule can be obtained upon the recombination of the primary radicals ^•^CHO and HN=C^•^H, which arise in the reactions of ^•^OH and ^•^H with CO, HCHO, and HCN (reactions 14, 16–19):

HN = CH^•^ + ^•^CHO + H_2_O→ NH_2_-CH_2_-COOH (23)

The ease of formation accounts for the predominant presence of glycine in various protocols of implementation of prebiotic chemistry. This amino acid, apparently, was the starting compound in the synthesis of other α-amino acids which form the polypeptide chain in protein biosynthesis. The general α-amino acid structure NH_2_-RCH-COOH includes a glycine moiety; the backbone radical NH_2_-^•^CH-COOH which is common to different amino acids, and the radical R^•^, which is different for various α-amino acids. The radical NH_2_-^•^CH-COOH appears upon radiation-induced reactions of glycine with H^•^ [83] and ^•^OH [84]:

^•^H + NH_2_RCHCOOH → NH_2_^•^CHCOOH + H_2_      7.1 × 10^4^ (24)

^•^OH + NH_2_RCHCOOH → NH_2_^•^CHCOOH + H_2_O        1.7 × 10^7^ (25)

Further, it can be assumed quite reasonably that simple α-amino acids are formed via the addition of radicals R^•^ to the “glycine” radical NH_2_-^•^CH-COOH in the α-position:

R^•^ + NH_2_-^•^CH-COOH → NH_2_-RCH-COOH (26)

The radicals R^•^ present in simple amino acids, alanine and serine, were formed in the primary reactions of “volcanic” gases with the radical ion products of water radiolysis. These are radicals ^•^CH_3_ (reaction 9) and ^•^CH_2_OH (reaction 22), respectively. Other amino acids are generated in several steps via reactions of organic molecules. For obtaining the radicals R^•^ present in threonine, cysteine, asparagine, glutamine, and aspartic and glutamic acids, it is necessary first to assemble the molecules CH_3_CH_2_OH, CH_3_SH, CH_3_CONH_2_, CH_3_CH_2_CONH_2_, CH_3_COOH, and CH_3_CH_2_COOH, respectively. More complex amino acids are formed under specific conditions, and apparently in the presence of enzymes. It is necessary to emphasize that the radiation-induced synthesis of amino acids in water does not require harsh conditions that were required in the Miller-Urey and other experiments [70,71,72,73,74,75,76,77,78,79,80]. The major difference is that organic synthesis takes place not in a gas-vapor environment at high temperature, but in a condensed medium (water) at any temperature.

The initial molecule for the radiation-induced formation of sugars is, probably, carbon monoxide CO. Like HCN, the carbon monoxide molecule has a triple bond. At room temperature, CO is non-reactive, but the reactivity markedly increases on heating and in solutions. However, the situation is sharply different when CO reacts with radicals. In this case it is highly reactive and, such as HCN and other unsaturated compounds, tends to add radicals to give adducts. When CO reacts with oxygen, carbon dioxide CO_2_ is formed. However, the lack of free oxygen in the early period allowed CO to react with other species in the hydro- and atmosphere of the planet. Reactions (10, 16, 19) illustrate the involvement of CO in chemical reactions with radical species *e*_aq_^−^, H^•^, and ^•^OH in water. It is known [85] that HCHO is formed in aqueous solutions containing CO and/or CO_2_ on exposure to radiation. In other words, this ancient abiogenic organic molecule also appeared, most likely, in the early Earth in sea water, for example, via the reduction of CO with ^•^H according to reactions (16) and (27), and with *e*_aq_^−^ according to (19) and (28)

^•^CHO + ^•^H → HCHO (27)

^•^CHO + *e*_aq_^−^ (H^+^) → HCHO (28)

The condensation of formaldehyde is a well-studied reaction [86]. In aqueous solutions, formaldehyde polymerizes at relatively high concentrations (≥10^−3^ mol L^–1^) to give sugars of various complexities, including ribose and/or deoxyribose. In our opinion, polymerization in sea water follows a radical mechanism where formaldehyde adds to the ^•^CHO radical to give successively radical fragments of glycolaldehyde, triose, tetrose, and, finally, ribose ^•^C_5_H_9_O_5_:

^•^CHO + 4HCHO → ^•^C_5_H_9_O_5_ (29)

This mechanism implies the possibility of condensation of HCHO, present in a low concentration in solution. The radical nature of ribose and deoxyribose ^•^C_5_H_9_O_5_ ensures high chemical reactivity for the subsequent participation in the synthesis of not only ATP, but also DNA and RNA in the molecules of which it is also present.

This assumption is confirmed by the fact that HCHO does, indeed, arise in aqueous solutions containing CO, on exposure to radiation. According to [85], HCHO, glyoxal H_2_C_2_O_2_, HCOOH (in 0.05, 0.03, and 0.04 µmol J^−1^ yields), and CO_2_ are formed in acidic CO solutions (4.8×10^−4^ mol L^–1^). The first step is the addition of ^•^OH and ^•^H to CO to give ^•^CO_2_H (k = 2×10^9^ L mol^–1^ s^–1^) [59] and ^•^CHO (k = 3.3×10^7^ L mol^–1^ s^–1^) [83], which further recombine. That is, formaldehyde and the simplest product of its condensation (glyoxal) are actually formed upon the radiolysis of an aqueous solution of CO. This fact provides a reasonable conclusion that the oldest abiogenic organic HCHO molecule also, most likely, appeared on the early Earth in sea water in the indicated CO reactions with radical products of water radiolysis. The subsequent polymerization of HCHO affords sugars of various complexity. The condensation of HCN proceeds, apparently, by a similar radical mechanism involving the intermediate HCN^•−^ (or HN=C^•^H) radical, and gives rise to the adenine ^•−^C_5_H_4_N_5_ radical:

HCN^•−^ + 4HCN → ^•−^C_5_H_4_N_5_ (30)

**Scheme 1:**
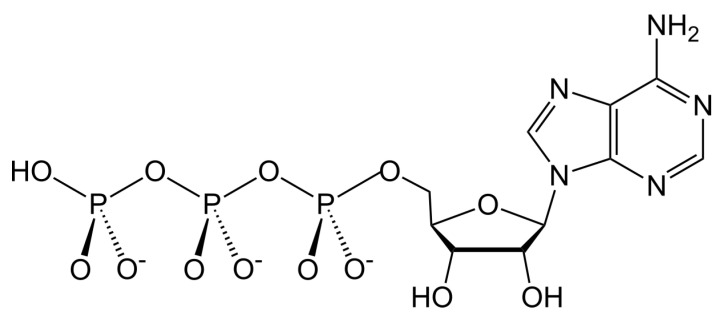
Structure of ATP.

An important role in biological processes belongs to adenosine triphosphate – ATP (Scheme 1). It is a versatile source of free energy participating in all biochemical reactions that absorb energy, such as formation of enzymes. ATP is composed of three parts: a nucleic base (adenine), a sugar (ribose), and phosphate groups.

The ATP molecule, being involved in a biochemical reaction, gives off energy as a result of hydrolysis to adenosine diphosphate (ADP) or adenosine monophosphate (AMP) and phosphate groups:

ATP + H_2_O → ADP (or AMP) + H_3_PO_4_ (or 2 H_3_PO_4_) (31)

Probably, the possibility of abiogenic synthesis of this molecule, at an early stage of evolution of the Earth’s matter, predetermined the emergence of life. It can be seen that the combination of the ribose ^•^C_5_H_9_O_5_ and adenine ^•−^C_5_H_5_N_5_ radicals produces the ATP backbone. The phosphate group completes the ATP molecule.

The radical mechanism of formaldehyde and hydrogen cyanide condensation explains the selectivity of this process, and the possibility for the process to occur at low concentrations of HCHO and HCN in the ocean. Indeed, the ^•^CHO and HCN^•−^ radicals catalyze the addition of unsaturated HCHO and HCN (reactions 29 and 30) to yield ribose and adenine, respectively. Adenine and the phosphate group are also parts of DNA and RNA. Apart from adenine, DNA has three more bases—thymine C_5_H_6_N_2_O_2_, guanine C_5_H_5_N_5_O, and cytosine C_4_H_5_N_3_O, and ribose is replaced by deoxyribose. RNA has the same structure and composition as DNA, except that the sugar is ribose, and thymine is replaced by another base, uracil. Presumably, the mechanism of formation of DNA and RNA fragments is generally the same as that considered above in relation to ATP formation. In other words, the principle of formation of larger groups, by a combination of radical groups that arose in radiation-induced reactions, is preserved. It is clear that the radiation mechanism is just a part of a complex and multistage process of the evolution of Earth’s matter, which ended in the appearance of life. It is noteworthy that relying on the proposed radical mechanism of the formation of adenine and sugar by polymerization of HCN and HCHO, the formation of the cytosine molecule C_4_H_5_N_3_O can be interpreted as a “mixed” condensation of three HCN molecules, and one HCHO molecule. It is important to emphasize that the stationary character of the radiolytic synthesis, over hundreds of millions of years, ensured the continuous production of amino acids, DNA, RNA, and ATP fragments and, as a result, the evolutionary nature of the ordering of organic molecules.

## 6. Formation of Organic Matter and Purification of the Ocean

The presence of HCN, CO, and other compounds with unsaturated groups (HCNO, HCONH_2_, H_2_NCN, (CN)_2_, HCSN, and many others) in water, with their proneness to polymerization and condensation under irradiation, was apparently responsible for the transformation of the Earth’s inorganic matter into organic matter. These compounds acted as active sites that initiated the formation of condensed matter. In the aqueous medium, the molecules were selected according to their water solubility and reactivity towards the radical ion products of water radiolysis; this selection allowed for the subsequent chemical transformations by a radical mechanism. Thus, the presence of HCN, (CN)_2_, HCHO, and CO molecules in the atmosphere and hydrosphere—even in very low concentrations—and the acting selectivity mechanisms in place, ensured their involvement in the chemical reactions in sea water, and accumulation of products with time. 

The possibility of radiation-induced formation, in the ocean, of organic molecules included in important biochemical processes, cannot be interpreted as the origin of life on the Earth. It can only be reasonably argued that the conditions on the primitive Earth were favorable for radiation-induced chemical reactions that resulted in the formation of racemic mixtures (containing both L and D enantiomers) of complex organic compounds from simpler inorganic precursors. Nevertheless, these organic molecules could probably serve as the building material for the fabrication of more complex “biomolecules”, and act as prebiotics of simple bacteria.

The radiation-induced chemical reactions of inorganic compounds dissolved in sea water promoted the implementation of two interrelated processes important for evolution: formation of organic matter, and purification of the ocean from toxic impurities. In turn, purification of the ocean was favorable for the formation of an environment that enabled the origin of life. The formation of organic compounds via transformation of inorganic matter of the Earth contributed to the same goal. The fact that the first signs of simple organisms were found to exist 4 Ga ago [5,6,7,8,9,10] indicates that the transformation of matter has actively proceeded, even during the planet’s formation and then during the formation of the ocean, i.e., in the first 500 million years (Hadean). Unfortunately, there are virtually no reliable data on the geochemical state of the early Earth. There are two points of view on the composition of the primitive atmosphere: (1) it mainly consisted of CH_4_, CO, and NH_3_, i.e., the atmosphere was reducing; (2) carbon mainly existed as the dioxide CO_2_, i.e., the atmosphere was oxidative [1,2,3,4]. It was noted above that the simplest amino acid, glycine, which served as the basis for the formation of other vital amino acids, was most likely generated upon the reaction of ^•^H with CO and HCN. Additionally, ribose and adenine, which are the major parts of ATP, DNA, and RNA, were produced by the condensation of HCHO and HCN, respectively. In our opinion, these facts most likely provide evidence in favor of the reducing atmosphere on the early Earth. Analysis of possible reactions shows that HCN, CO, and HCHO had a significant predominance over many other compounds that would be expected to appear in the early ocean. This follows on from the relatively high-rate constants for the reactions of these molecules (10^7^–10^9^ L mol^−1^ s^−1^). For example, CO_2_ and NH_3_ exist in water as CO_3_^2−^ and NH_4_^+^, which have low reactivity towards e_aq_^−^, ^•^H, and ^•^OH (≤10^4^ L mol^−1^ s^−1^). Therefore, CO, and HCHO should be present in approximately 10^3^–10^5^ higher concentrations than HCN to be competitive with it. That is, the presence of HCN, CO, and HCHO in water, even in rather low concentrations, gives them a pronounced advantage for reactions with e_aq_^−^, ^•^H, and ^•^OH, and hence for the subsequent involvement in the formation of amino acids, sugars, and nucleic bases. One more specific feature of radiation-induced reactions of these molecules is the ability of ^•^CHO and HCN^•−^, derived from these molecules, to act as condensation centers and to initiate radical polymerization, giving rise to macromolecular products. These substances are separated into an insoluble phase and are removed from the area under irradiation. Thus, the continuous supply of HCN, CO, and HCHO into sea water should have ensured the transformation of the inorganic matter of the Earth, into organic matter. In addition to HCN, cyanogen (CN)_2_ might also play an important role in the radiolytic transformations leading to the formation of organic matter. Like HCN, cyanogen has the reactive −C≡N group, and therefore it can be readily transformed in the radiation-induced chemical reactions into organic compounds, including polymers. In addition, cyanogen is hydrolyzed in water to give HCN and cyanic acid HOCN (or isocyanic acid HNCO). All compounds containing a −C≡N group with an unsaturated bond tend to undergo radiation-induced chemical reactions to give organic amino compounds, and polymers. These compounds accumulate various radicals that arise upon radiolysis, and act as centers of formation of condensed matter. Table 2 gives the reactions and rate constants for reactions of *e*_aq_^−^, ^•^H, and ^•^OH with some carbon- and nitrogen-containing compounds. These compounds were present among volcanic gases, or were formed in the early atmosphere via chemical reactions. Most likely, they were the initial species for the formation of organic matter.

Another important circumstance that promoted the radiolytic transformation of HCN and (CN)_2_, as well as HCHO, into biological compounds is, as indicated above, their high solubility in water. Therefore, they had preference in the migration from the atmosphere to sea water. Indeed, HCN and HCHO are infinitely soluble in water, while the volume coefficient of solubility (mL of a solute in 100 g of water) for (CN)_2_ is approximately 450. The solubility of gases O_2_, H_2_, CO, CH_4_, and N_2_ is in the range of 1.5–3.5. This was a criterion for the selection of molecules, promising for prebiotic chemistry, from the atmosphere to the hydrosphere. In water, molecules were selected in terms of their reactivity towards the radical and ionic products of water radiolysis, which provided their subsequent chemical transformations by the radical mechanism. Finally, the unsaturation of the −C≡N group and the C≡O molecule enabled their subsequent polymerization to give organic matter, which formed a separate phase. It was shown by pulsed radiolysis [98] that Cyanide-H^•^ (HN=^•^CH adduct) and Cyanide-^•^OH (HOCH=N^•^ adduct) recombine, with the reaction rate constants being very high: ~1.4 × 10^9^ L mol^−1^ s^−1^. High reactivity of radicals with CN groups, and their ability to act as condensation centers for compounds present in the ocean, was also demonstrated in relation to cyanic acid HCNO [99]. The Cyanate-^•^OH adduct formed in the reaction (see Table 2):

NCO^−^ + ^•^OH → ^•^NC(OH)O^−^ (32)

was shown to tend to add additional OCN^−^ ions, i.e., it is able to act as a center for radical chain polymerization

^•^NC(OH)O^−^+ NCO^−^→ ^−^OC(OH)NNC^•^O^−^        4.3 × 10^6^ (33)

The cyanate radical ion ^−^OC(OH)NNC^•^O^−^ thus formed is also highly chemically reactive towards various organic compounds. The rate constants of its reaction with the ascorbate ion, hydroquinone, methoxyphenol, phenylenediamine, tetramethyl-*p*-phenylenediamine, and urate ion are approximately (7 × 10^7^–4 × 10^8^) L mol^−1^ s^−1^, while the rate constants for the reactions with aniline and phenol are <5 × 10^6^ L mol^−1^ s^−1^. There is a tendency for radical transfer to these compounds. Thus, the action of radiation on aqueous solutions containing CN compounds, first, initiates their polymerization and, second, makes them react with other organic compounds according to a chain mechanism. A similar behavior of the CO molecule is evidenced by the data on the formation of formaldehyde, the product of its primary condensation (glyoxal), and more complex sugars upon the radiolysis of an aqueous solution of CO [85].

Thus, the appearance of HCN, (CN)_2_ and HCHO, CO molecules in the atmosphere and the hydrosphere, even in a very low concentration, provided the subsequent accumulation of the products they form with time. The most important factor in this process was the stationary character of the radiation, which ensured the evolutionary changes of molecules, and accumulation of organic matter over hundreds of millions of years.

Hydrogen cyanide, cyanide salts, and cyanogens are highly potent poisons. Other compounds containing a cyano group –C≡N (or =C=N) are also toxic. In particular, this refers to organic compounds—nitriles and isonitriles, cyanic and isocyanic acid and their derivatives, and many others. Toxicity is also inherent in CO and HCHO. It is amazing that Nature chose these toxic compounds to design the molecules of life. However, high reactivity of these compounds apparently implied the possibility of their easy degradation on exposure to radiation. The decay of natural radioactive isotopes initiated their degradation, and hence this promoted the radiation purification of ocean water from toxins. Note also the proneness of cyano derivatives to hydrolysis, giving organic acids and ammonia, which is thus accompanied by the loss of toxic properties of cyano compounds. The accumulation of organic matter took place gradually over a long period of time, and simultaneously purification of the ocean took place. The conditions in the aquatic environment were thus prepared for the subsequent origin of life. The efficiency of radiation purification of water was recognized to its full extent only nowadays. Now the use of radiation for water purification, from toxic impurities, is considered to be one of the most efficient and promising methods [58,102,103,104]. The radiation treatment of water refers to advanced oxidation technologies (AOP technologies). High penetrating power of radiation ensures the destruction of both dissolved, and suspended impurities. The purifying effect of radiation is due to its ability to inactivate toxic and chromophore functional groups, transform impurities into an easily extractable form, damage the DNA of microorganisms and their spore forms, and increase the biodegradability of organic impurities. The concentrations of radical ion products (of the order of decimols per liter), which are attained upon sea water radiolysis, are markedly higher than the concentrations of impurities (about ~10^−7^–10^−3^ mol L^−1^) that could be expected to be present in water in the early stage, as a result of volcanic activity (methane, ammonia, carbon oxides, etc.). The radiation-induced transformations of sea water affect the chemical composition of water. The presence of transition metal ions and organic compounds—as well as the appearance and accumulation of oxygen (see the next chapter)—may enhance the radiation effect of chemical transformations of dissolved compounds by a large factor, by initiating chain reactions involving radicals [58].

## 7. Quantitative Evaluation of the Formation of Organic Matter

Reliable information on the amounts of radioactive isotopes on the Earth since its formation, and the radiation energy released upon their decay, enables a rational evaluation of the formation of organic matter from inorganic matter by the radiation mechanism. This synthesis is related, first of all, to the involvement of unsaturated molecules (HCN, (CN)_2_, CO, HCHO, and other) and the products of their reactions in the successive condensation and polymerization reactions initiated by the radical ion products of water radiolysis. The inorganic to organic transformation also involved other compounds present in the ocean (CH_4_, NH_3_, CO_2_, H_2_S, inorganic acid anions, and other). The process ended in the isolation of the organic matter as a separate phase. The chain mechanism includes successive initiation (34), chain propagation (35), and chain termination (36) steps:

M + e_aq_^−^ (or ^•^H and ^•^OH) → M^•^ (34)

M^•^ + (n−2)M → M^•^_n−1_ (35)

M^•^_n−1_ + M^•^ → M_n_ (36)

For quantitative estimation, it is necessary to use the chain propagation number n, which is unknown. Therefore, we assume that the radiation-initiated condensation of molecules M occurs only as “fusion” of radicals M^•^, resulting from the reaction of molecules M (i.e., HCN, (CN)_2_, HCHO, CH_4_, and other) with e_aq_^−^, ^•^H, and ^•^OH, according to Equations (9)–(22) indicated in Table 2. Thus, we have

(x − 2)M^•^ → M^•^_x−1_ (37)

M^•^_x−1_ + M^•^ → M_x_ (38)

The difference between the mechanisms described by reactions (34–36), on the one hand, and reactions (37, 38), on the other hand, is as follows. According to the former mechanism, each water-derived radical (e_aq_^−^, ^•^H and ^•^OH) consumes n molecules M for the chain formation of organic matter, while in the latter mechanism, it is only one molecule M. Since the n value is unknown, we use the latter mechanism of the formation of organic matter in the calculation. This calculation gives markedly lower yields of organic matter. We take the molecular weight of M to be 30, which is approximately the average of the molecular weights of indicated HCN, (CN)_2_, HCHO, and other molecules. We assume that the final molecule M_x_, which has a weight of approximately 300–1000, i.e., contains 10–30 molecules M, is isolated from the aqueous solution as a separate phase and no longer participates in the radiation-induced chemical reactions. This results in accumulation of organic matter. Further, we assume that all radical products of water radiolysis are captured by the dissolved compounds. Then, the amount of organic matter formed in the ocean upon the decay of the radioactive ^40^K isotope alone is described by the equation

**Figure 4:**
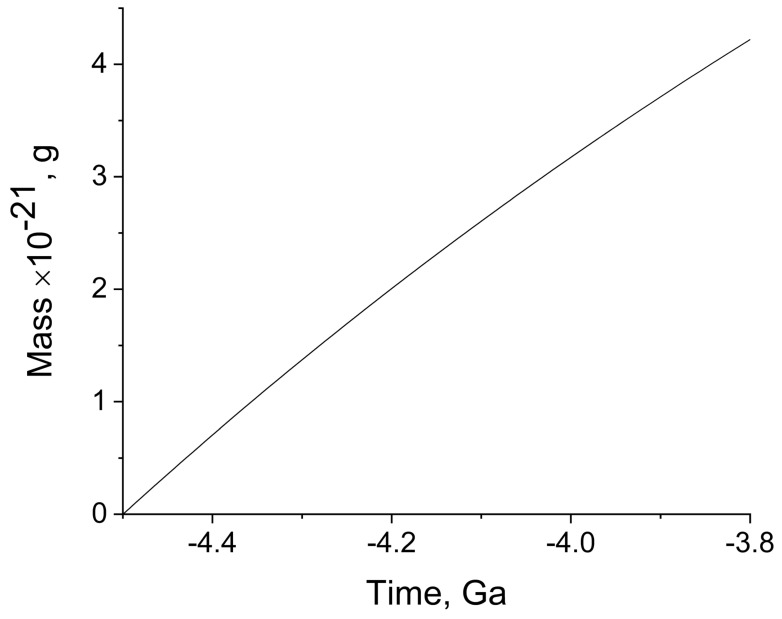
Accumulation of organic matter in the ocean upon the radiation-induced transformation of dissolved inorganic substances.

[Pr]_t_ = [C_K_ × A_K_^−1^ × E × G(Pr) × M_H2O_^−1^ × 10^−2^] ×(1 − e^−λt^) (39)

where C_K_ is the amount of ^40^K in the ocean 4.5 Ga ago (g); A_K_ is the atomic weight of potassium (g); E is the average energy released during the decay of a ^40^K atom (5.9×10^5^ eV); G(Pr) is the total radiation-chemical yield of ion-radical products of water radiolysis eaq- (0.28 µmol J^−1^), ^•^H (0.6 µmol J^−1^), and ^•^OH (0.28 µmol J^−1^), equal to 0.62 μmol J^–1^ (see Equation (3)); M_H2O_ is the amount of water in the ocean involved in the radiation chemical synthesis (1.4 × 10^21^ kg). Figure 4 illustrates the build-up of the weight of organic matter caused by water radiolysis, and induced by the decay of ^40^K alone, during the early period of Earth’s existence (Hadean) characterized by volcanic and tectonic activity. After this period, in the beginning of the Archean (4.0–3.8 Ga), the simplest anaerobic forms of life already appeared and developed.

It can be seen that the accumulation of mass during the short Hadean period, followed nearly linear time dependence, and in the period from 4.5 Ga to 4.0 Ga, approximately 3 × 10^21^ g was formed. The contribution of ^235^U; ^238^U; and ^232^Th was markedly lower; these isotopes increased the indicated yield of organic matter by approximately 30%. The mass of organic matter currently present on the Earth, including all representatives of flora and fauna, is roughly estimated to be 10^18^ g. However, this matter continuously forms in various processes, degrades, and forms again. On the early Earth, organic matter also existed in the same equilibrium state between formation and degradation. Therefore, its steady-state amount was always significantly lower than that obtained in our calculations, for the whole period of 500 million years. If we take the degradation and restoration period to be, for example, 100 years, the steady-state amount of organic matter that was permanently present on the Earth between 4.5 and 4.0 Ga ago is found to be approximately 6 × 10^14^ g. This amount is three to four orders of magnitude lower than the amount of organic matter on today’s Earth. The difference is quite understandable and explainable, in view of the fact that at present, organic matter is formed upon photosynthesis involving oxygen, and is mainly composed of plants. On the early Earth, there was little organic matter, and its appearance and mass build-up implied the coming of the planet to life. The organic molecules were gradually converted to the simplest living species along the obscure paths of evolution. 

## 8. Oxygenation of Hydro- and Atmosphere

As the volcanic activity was attenuated and the Earth cooled down, the composition of the Earth’s atmosphere gradually changed. A crucial change in the atmosphere, in which volcanic gases were replaced by oxygen, took place approximately 2.4 billion years ago due to the development of oxygen photosynthesis by blue-green algae (cyanobacteria). This brought about mass extinction of previously existing anaerobic life forms, and the spread of energetically more favorable oxygen-breathing species, i.e., GOE, took place [1,2,3,4,19,20,21,22,23]. The transition from anaerobic fermentation to oxygen respiration was gradual and required the presence of free oxygen in a relatively low concentration in the atmosphere, even in the prebiogenic period of Earth’s development. Presumably, only in this case, cyanobacteria could appear and switch to the photosynthetic assimilation of carbon dioxide, and production of oxygen. Indeed, according to geochemical studies [20,24,25,26], a minor amount of oxygen was present in the atmosphere even at the very early stages of Earth’s existence, since around 3 billion years ago or even earlier. Perhaps, the laminated iron ores detected in the south-western part of Greenland attest to the early oxygenation of the atmosphere (approximately 3.8 billion years ago) [46]. The formation of such ores requires the presence of free oxygen to oxidize divalent iron to the trivalent state. In other words, even at that time, there were some sources of oxygen supplied to the exosphere. Water and carbon dioxide photodissociation, in the upper layers of the Earth’s primary atmosphere, is considered as the most probable (external) source [105,106,107]. Early oxygen photosynthesis, as a source of oxygen, cannot be ruled out either.

In our opinion, in the early stages of the existence of Earth, free oxygen was mainly generated by natural radioactive isotopes [29,30,31,32]. As indicated above (Section 3.2), their radioactive decay initiated water splitting to give oxygen. Molecular oxygen is not a primary product of water radiolysis, it is formed via decomposition of hydrogen peroxide H_2_O_2_ (reaction 4). According to the mass balance of decomposition of a water molecule

**Figure 5:**
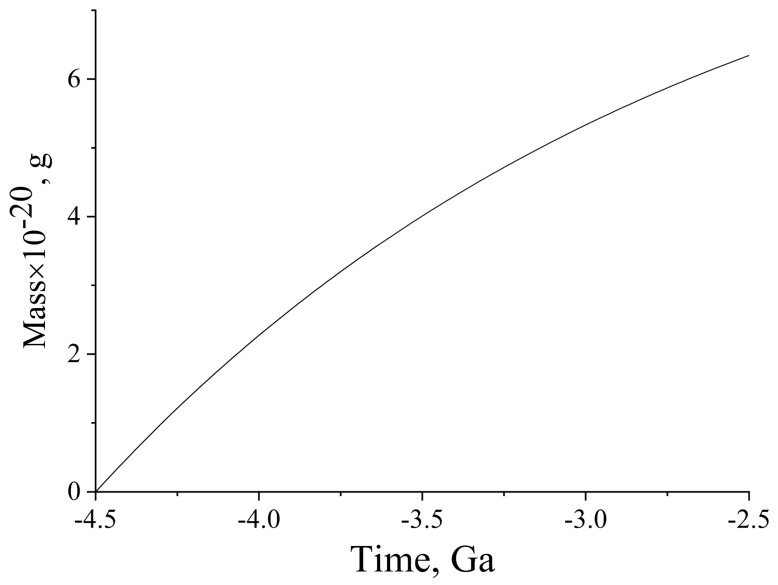
Time dependence of oxygen formation upon radiolysis of sea water.

H_2_O → H_2_ + 1/2 O_2_ (40)

the radiation yield of O_2_ should be equal to the half of the yield of H_2_, i.e., for γ-ray or β-particles, it is approximately 0.022 μmol J^–1^, while for α-particles, it is about 0.07 μmol J^–1^ (see Section 3.2). The oxygen formation upon water radiolysis and determination of the radiation chemical yield were discussed in detail previously [29,30]. The time dependence of oxygen accumulation in the atmosphere under the radiation of ^40^K and ^235^U, ^238^U in the ocean is shown in Figure 5.

The major contribution to oxygenation is associated with the decay of ^40^K. The contribution of ^235^U and ^238^U is approximately 25% of the total amount. It can be seen that the decay of isotopes could give rise to approximately 6.2 × 10^20^ g of O_2_ entering the hydro- and atmosphere of the early Earth, in the period between 4.5 Ga ago and GOE (2.4 Ga ago). This amount is comparable with its current content in the atmosphere (1.2 × 10^20^ g). Thus, the natural radioactive isotopes ^40^K, ^235^U, ^238^U, and ^232^Th could serve as the internal source of energy that provided continuous oxygenation of the hydro- and atmosphere of the early Earth, as a result of ocean water radiolysis. 

The oxygenation and the evolution of life are linked by several threshold points. It is generally accepted that the formation of oxygen was caused by the photodissociation of H_2_O and CO_2_ in the upper layers of the Earth’s primary atmosphere, under hard UV radiation from the Sun [105,106,107]. With this oxygenation mechanism, the accumulation of O_2_ cannot exceed the threshold value, equal to 0.001, of the current oxygen content of 1.2 × 10^21^ g (Urey point). The concentration of 1.2 × 10^18^ g should be maintained by itself because of absorption of UV radiation by the formed oxygen. That is, as this concentration has been attained, oxygen starts to shield further decomposition of water by the photochemical mechanism. With this oxygen content, only anaerobic life could exist on the Earth. It is obvious that the external source does not affect the oxygen formation from the internal source (radioactive isotopes). With the radiation-induced degradation of sea water, the threshold concentration of oxygen (Urey point) is attained rapidly on the geological time scale, within approximately 4–5 million years. The next threshold point (Pasteur point) is attributed to the possibility of appearance and existence of oxygen-breathing living cells. This corresponds to oxygen content in the atmosphere equal to 0.01 of the current level, that is, 1.2 × 10^19^ kg. This oxygen level is favorable for the appearance of organisms that can reversibly switch their energy metabolism from respiration to fermentation, as the oxygen content varies in the vicinity of the Pasteur point. The restriction of the development of oxygen-breathing life is due to the deleterious action of UV radiation, caused by the weak ozone shield. With the radiation mechanism of oxygenation, this amount of oxygen should have been formed in approximately 40–50 million years, i.e., this point also could have been crossed in a very early period of the Earth’s existence. Finally, the third threshold amount of oxygen in the atmosphere (the Berkner-Marshall point [108,109]) corresponds to 0.1 of the current level, i.e., 1.2 × 10^20^ g. This content of oxygen enables the formation of the protective ozone shield to preserve oxygen-breathing life. It can be seen in Figure 5 that this oxygen content in the atmosphere was attained 4.25 Ga ago. In other words, the threshold amount of oxygen that allows for the development of oxygen-breathing life (Berkner-Marshall point) could have been attained long before GOE. These considerations are purely speculative, because they refer to the unlikely situation in which oxygen is formed, but not consumed. Certainly, this is not the case. Actually, the rising oxygen, and most likely also H_2_O_2,_ its precursor, were consumed in diverse reactions such as oxidation of iron and other metals, formation and decomposition of inorganic and organic compounds, and a multitude of other reactions. An important consequence of the presence of natural radioactive isotopes in sea water was the existence of a continuous, and powerful internal source of energy. This source could provide the supply of oxygen to the Earth’s hydro- and atmosphere; thus it could bypass the indicated barriers (threshold points) to the oxygen and ozone shielding of energy supply from an external source (Sun). Over time, sea water was purified and spots with elevated oxygen content appeared, and hence oxygen consumption in biochemical reactions became possible. Therefore, the situations corresponding to the Urey, Pasteur, and Berkner-Marshall threshold points should be considerably shifted towards the present time. However, it is obvious that the natural radioactivity on Earth is an important source of oxygen in the early stages of the Earth’s existence, and an important factor promoting the formation of the oxygen atmosphere of the Earth. 

In the case of an external source of oxygenation (Sun), oxygen gets into the ocean from the atmosphere, or is formed in a thin near-surface active zone. This means that the oxygen concentration gradient is directed from the atmosphere towards the ocean depth, i.e., the outer layers may be saturated, while no oxygen may be present in deep-water. The radiation-induced oxygenation follows an entirely different pattern. Apparently, oxygen arises uniformly throughout the ocean bulk, which corresponds to the uniform distribution of the dissolved radioactive isotopes. As the saturation is reached, oxygen moves upwards, and migrates to the atmosphere. This mechanism implies the possibility of retardation in the oxygen saturation of the atmosphere, thus highlighting the essential lag between atmospheric and oceanic oxygenation, and setting the stage for a generation of research in Precambrian oxygenation. This delay, before the atmospheric oxygenation, could be enhanced by slow oxygen diffusion from the ocean depth at a high pressure of water mass. Oxygen appeared throughout the ocean bulk and was simultaneously consumed in a variety of redox reactions. This should have resulted in a stationary, although relatively low, level of oxygen in seawater for tens and hundreds of millions of years. This was favorable for the appearance, and then development, of oxygen-breathing microorganisms in the ocean. 

## 9. Isn’t the Role of Natural Radioactivity Exaggerated? 

The substantiation of the hypothesis about the important role of natural radioactivity in the chemical evolution and formation of life on the primitive Earth, presented in this paper, is based on quite obvious and reliable facts. These facts include (i) the presence of radioactive isotopes since the Earth’s origin, and the amounts of the isotopes; (ii) types of decay, and the released energy of ionizing radiation; (iii) the mechanism of water radiolysis, and yields of the radical or ionic and molecular products; and (iv) particular radical reactions of compounds dissolved in water on the early Earth, and the measured rate constants for these reactions. In the calculations, we assumed that the amount of water on the planet was constant, and equal to that in the currently existing ocean. This corresponds to the modern views on the appearance of water by accretion of chondritic meteorites during the formation of Earth. Moreover, as indicated in Section 3.2, the amount of water on the Earth is 1.5–2.0 times greater than the amount of water in the ocean. In other words, the amounts of organic matter and oxygen formed upon the radiolysis of water, and aqueous solutions of compounds present in the atmosphere and geosphere of the early Earth, may well be higher. The quantitative estimate of the chemical transformation of matter of the early Earth, made in the present study, should be certainly regarded as underestimated. The calculations considered the ocean to be the only source of the evolutionary process. Meanwhile, the mass of ocean water is 5% of the mass of Earth’s crust, and the content of ^40^K (the main source of radiation) in the ocean is less than 0.06% of the total amount of ^40^K on the Earth (the greater part of ~77% is in the crust). Therefore, the radiation effect of the transformation of the matter in the crust can be more pronounced than that estimated only for the ocean. This is indicated by active microbial life deep below the Earth’s surface. There are reasons to believe that molecular hydrogen and oxidizing agents, formed as a result of water radiolysis, are a constant source of energy for these microbial communities [110,111,112]. However, the chemical evolution of molecules in the earth’s crust, apparently, was significantly limited in comparison with the ocean due to the difference in the phase state of the media. The radiation-induced decomposition of rocks requires special investigations, and therefore it is beyond the scope of the current discussion. It can be concluded, with confidence, that the calculations were performed at the minimum level without making any doubtful assumptions, and were based on known and reliable parameters. The proposed hypothesis of the important role of natural radioactivity and radiation-induced chemical reactions in the chemical evolution of the early Earth is certainly sufficiently substantiated, and has prospects for further development.

## 10. Conclusions

It is clear that an internal potent source of energy for chemical transformation of inorganic to organic matter, that is, natural radioactive isotopes (^40^K; ^235^U; ^238^U, and ^232^Th), has always existed on the Earth. The molecules that formed the first prokaryotic and eukaryotic living organisms arose in the atmosphere under harsh conditions, and under the action of solar radiation, plasma, electrical discharges, impacts of falling meteorites, and the heat of the Earth. Then, they were washed into the ocean. However, the ocean was not only a ***reservoir*** for these compounds, but also an efficient ***converter*** for their subsequent reactions. Particularly in the ocean, the key stage of the prebiotic chemistry took place, most likely. Natural radioactive isotopes served as the source of energy for the synthesis and accumulation of organic matter in the ocean. The potential of radiation-induced synthesis is vividly demonstrated by the mentioned experiment using a CO solution [85]. It is hardly possible to choose a simpler chemical composition. Nevertheless, radiolysis resulted in a variety of products: CO_2_, HCHO, glyoxal, CHO CHO, and HCOOH. Other products were present in amounts below the level of analytical determination. However, the formation of formaldehyde and glyoxal (as the first formaldehyde condensation product) reasonably suggests the possibility of subsequent formation of various sugars and alcohols, with a continuous supply of CO and irradiation. Abiotic organic synthesis based on radiolytic transformations of CO_2_ and HCO_3_^−^ and CO_3_^2-^ anions in water is much less efficient. The absence of unsaturated bonds in them affects this. However, in this case, the formation of formate and oxalate is fixed also [113,114,115,116,117,118,119]. These low molecular weight carboxylate compounds, along with H_2_, can play an important role in maintaining subsurface lithoautotrophic microbial ecosystems. The efficiency of radiation-induced synthesis is also supported by computer simulation of the radiolysis of simple organic compounds—oxalic and acetic acids. It was shown that the decomposition of oxalic acid gives tartaric, tartronic, glyoxylic, and many other organic acids [113,114], while the decomposition of acetic acids gives glycolic and glyoxalic acids, formaldehyde, and other products [115]. It can be stated, with sufficient certainty, that the radiation-induced chemical transformations of aqueous solutions of CO, CH_4_, HCHO, HCN, and other simple organic compounds makes it possible, in principle, to synthesize an almost infinite set of organic products. Necessary conditions are continuous supply of the starting compounds, and exposure to ionizing radiation, i.e., the conditions that existed on the early Earth. The accumulation of organic compounds in the ocean gave rise to an “organic soup”, in which additional reactions, and the formation of more complex organic molecules took place for hundreds of millions of years under the action of internal radiation, and external light and heat. The gradual shift of the place of synthesis from the toxic early atmosphere to the hydrosphere protected the arising molecules from the deleterious action of light, electrical discharges, and plasma; it also promoted further evolution of molecules during the decline of the active phase of the Earth’s development in the first hundreds of millions of years. It is noteworthy that the radiation stage occurred under stationary conditions of internal supply of radiation energy, and under greenhouse conditions of the ocean. The nutrition medium of prebiotic molecules needed for the simplest organisms was permanently reproduced in the aquatic environment. The simulation of radiation-induced reactions and experimental observations show that amino acids, sugars, fatty acids, and nitrogenous bases were formed in the ocean on the early Earth, under the action of indicated factors. There compounds are the building blocks that constitute the basis for all living forms nowadays, 4.6 billion years later. The radiation-induced splitting of water to give oxygen-ensured oxygenation of the hydro- and atmosphere. There are also no grounds to rule out the possibility that some organic compounds arrived on the early Earth from outer space. This is evidenced by the discovery of many molecules, including amino acids, bases, and fatty acids in meteorite fragments (1969, Australia, Murchison), and the presence of complex organic carbon compounds in carbonaceous chondrites [120]. Apparently, outer space could have been an important source of organic matter coming to the Earth in the early stage of formation of the solar system. The size and the surface of the ocean have changed over billions of years, which promoted redistribution of organic matter on the Earth. There appeared shallow and well-warmed lagoons, open and dry places and, hence, new opportunities for the emergence and development of life. The performed calculations show that the radiation mechanism could account for the formation of significant amounts of organic matter and oxygen, as a result of water radiolysis in the ocean. The radical products of radiolysis of sea water, apparently, played an important role in the purification of water from toxic impurities, formation of various simple “biomolecules”, and transformation of inorganic matter of the Earth into organic matter. The radiation-induced transformations proceeded on the Earth non-uniformly, while the calculations were performed for average conditions over the whole Global Ocean. Indeed, different areas on the Earth’s surface and in the ocean were apparently characterized by different radioactivity levels, temperatures, salinities, contamination levels of sea water, and many other parameters. In other words, there could appear a “warm little pond...” in which “…a protein compound was chemically formed ready to undergo still more complex changes”, as was suggested by Darwin [22]. This suggests a non-uniform appearance and distribution of organic matter, including prebiotics and oxygen on the Earth, and accordingly the non-uniform appearance and development of life forms. One can reasonably assume the presence of compartments with an increased, but not detrimental, radioactivity level in which an internal (radiation) source of oxygen was continuously operating. In the early Archean period, most bacterial groups in an anaerobic environment did not generate oxygen following photosynthesis. In order to trigger this process, free oxygen was required in the prebiogenic stage of the Earth’s development. Only in the presence of free oxygen in the atmosphere were cyanobacteria able to switch to the photosynthetic assimilation of carbon dioxide, and production of oxygen. In the above-mentioned compartments, conditions were suitable for the modification of anaerobic forms of life and appearance of oxygen-breathing life forms, with participation of natural radioactive isotopes. Apparently, the very early radiation-induced oxygenation of the atmosphere and the ocean was only able to initiate the emergence of oxygen-breathing biological forms, but this oxygenation was not always high. This does not rule out the early origin of oxygen photosynthesis. The possibility of oxygenic photosynthesis appeared with the advent of cyanobacteria. They created the stable oxygen-containing atmosphere of the Earth. 

The proposed hypothesis implies further development and substantiation. In the current state, it should be considered as the starting point for the subsequent consideration of the important role of natural radioactivity in the general picture of chemical evolution of the Earth, and the appearance of life.

## Figures and Tables

**Table 1:** Long-lived natural radioactive elements in the earth’s crust and ocean and their characteristics.

Isotopes	T_1/2_, bn Years	Radiation	Energy, MeV	Specific Activity × 10^−4^, Bq g^−1^	Crust			Ocean		
					Mass × 10^−19^, g		Released Energy × 10^−30^, J	Mass × 10^−15^, g		Released Energy × 10^−26^, J
					4.6 Ga	Now		4.6 Ga	Now	
^235^U	0.713	αβ	48.306 * 6.671 *	8.014	2.973	0.034	0.661	810	68.5	0.56
^238^U	4.47	αβ	57.902 * 17.544 *	1.246	9.57	4.69	1.488	2.84	0.033	0.97
^232^Th	14.05	αβ	40.134 * 7.057 *	0.407	22.67	18.07	0.801	~3 × 10^−4^	~2 × 10^−4^	~1 × 10^−5^
^40^K	1.28	βγ	0.455 1.46	25.89	131.04	10.86	1.618	810	68.5	10.1

* Total energy released in the form of α- and β-particles, respectively.

**Table 2:** Rate constants of *e*_aq_^−^, ^•^H, and ^•^OH with carbon- and nitrogen-containing compounds.

Radical	Reaction	k, L mol^−1^ s^−1^	Reference
^•^H	HCN + ^•^H → HN=^•^CH	3.7 × 10^7^	[87]
	(CN)_2_ + ^•^H → products	<1 × 10^7^	[88]
	H_2_NCN + ^•^H → H_2_NCH=N^•^	6.9 × 10^6^	[89]
	N_3_^−^ + ^•^H → HN_3_^•–^	2.4 × 10^9^	[90]
	HN_3_ + ^•^H → products	7.2 × 10^7^	[91]
	SCN^−^ + ^•^H → products	2.3 × 10^8^	[92]
	CN^−^ + *e*_aq_^−^ → products	3 × 10^5^	[93]
*e*_aq_^−^	(CN)_2_ + *e*_aq_^−^ → (CN)_2_^•–^	2.1 × 10^10^	[88]
	HCONH_2_ + *e*_aq_^−^ → ^•^CONH_2_	2 × 10^7^	[94]
	N_3_^−^ + *e*_aq_^−^ → products	<1.5 × 10^6^	[95]
	HN_3_ + *e*_aq_^−^ → HN_3_^•–^	1.2 × 10^10^	[96]
	H_2_NCN + *e*_aq_^−^ → H_2_NCN^•–^	1.5 × 10^9^	[89]
	SCN^−^ + *e*_aq_^−^ → products	<1 × 10^6^	[97]
	HCN + ^•^OH → HOCH=N^•^	6 × 10^7^	[98]
^•^OH	CN^−^ + ^•^OH → ^•^C(OH)=N^−^	8 × 10^9^	[93]
	NCO^−^ + ^•^OH → ^•^NC(OH)O^−^	4.8 × 10^7^	[99]
	(CN)_2_ + ^•^OH → ^•^CNCNOH	<1 × 10^7^	[88]
	N_3_^−^ + ^•^OH → ^•^N_3_ + OH^−^	1.4 × 10^10^	[100]
	H_2_NCN + ^•^OH → H_2_NC(OH)=N^•^	8.7 × 10^6^	[89]
	SCN^−^ + ^•^OH → HOSCN^•–^	1.2 × 10^10^	[100]
	NH_3_ + ^•^OH → ^•^NH_2_ + H_2_O	9.7 × 10^7^	[101]

## Data Availability

Not applicable.
